# Demographics Regarding Belief in Non-Human Animal Sentience and Emotional Empathy with Animals: A Pilot Study among Attendees of an Animal Welfare Symposium

**DOI:** 10.3390/ani8100174

**Published:** 2018-10-04

**Authors:** Amelia Cornish, Bethany Wilson, David Raubenheimer, Paul McGreevy

**Affiliations:** 1 Sydney School of Veterinary Science, University of Sydney, Sydney, NSW 2006, Australia; bethany.wilson@sydney.edu.au (B.W.); paul.mcgreevy@sydney.edu.au (P.M.); 2 Charles Perkins Centre, University of Sydney, Sydney, NSW 2006, Australia; david.raubenheimer@sydney.edu.au; 3 School of Life and Environmental Sciences, University of Sydney, Sydney, NSW 2006, Australia

**Keywords:** attitudes to animals, animal sentience, animal welfare, cluster analysis, empathy with animals, pilot study

## Abstract

**Simple Summary:**

Attitudes towards the welfare of non-human animals are related to beliefs about animals’ capabilities, particularly regarding experiences of pain and suffering. The current study explores the attitudes to animals among people who attended an animal welfare symposium at the University of Sydney. This population represents a unique sample of people who work, study or have a strong interest in animal care and welfare. The pilot study used a validated questionnaire that assessed attitudes to animals; specifically exploring participants’ (*n* = 41) beliefs about the sentience of animals and their emotional empathy with animals. The results found significant associations between participants’ beliefs in animal sentience and their demographic variables (age, sex and occupation). Female attendees showed stronger belief in sentience than male attendees did. When looking at emotional empathy with animals, the participants’ responses to the statements assigned into three clusters (or distinct groups) based on their content which reflected the internal emotional lives of animals and the treatment of animals by humans (Cluster 1), human interactions with animals (Cluster 2) and the keeping of companion and zoo animals (Cluster 3).

**Abstract:**

Attitudes to animals are linked to beliefs about their ability to experience pain and suffering, their cognition, and their sentience. Education and awareness-raising play a pivotal role in increasing society’s consideration of non-human animal welfare. The current pilot study explores the attitudes towards animal welfare among a unique population of people who attended an animal welfare symposium at the University of Sydney. It involved administration of a validated questionnaire that assessed attitudes to animals; specifically exploring participants’ (*n* = 41) beliefs about the sentience of animals and their emotional empathy with animals. The resultant data revealed significant associations between participants’ beliefs in animal sentience and their demographic variables (age, sex and occupation). Female attendees showed stronger beliefs in sentience than male attendees did. Concerning sentience in cows, pigs and cats, older attendees showed stronger beliefs than younger people in sentience relating to hunger and pain. Also, with regard to questions about sentience in dogs, older attendees showed stronger beliefs than younger people in pain-related sentience in dogs. When exploring emotional empathy with animals, the participants’ statements could be assigned to three clusters characterised by the internal emotional lives of animals and the treatment of animals by humans (Cluster 1), human interactions with animals (Cluster 2) and the keeping of companion and zoo animals (Cluster 3). To the authors’ knowledge, this pilot study is the first of its kind to investigate the attitudes towards animal welfare of an important group of people who work, study or have a special interest within the animal care and welfare domain.

## 1. Introduction

Over the past few decades, traditional functional conceptions of animals as objects to be used by humans have increasingly given way to an ethic of care and compassion, as evidenced by increasing public sensitivity and concern about animal use [1,2,3]. Attitudes can be considered learned dispositions that help to explain behaviour [4]. Public attitudes to the use of animals have direct and indirect effects on the treatment of animals in the human domain. Furthermore, a better understanding of trends in public attitudes to animal welfare can provide crucial insights into the ways in which the public can be expected to respond to topical issues regarding animals in the community.

The term “animal sentience” refers to the notion that animals experience an array of feelings from pain and suffering to pleasure and joy [5]. Animals have been identified as “sentient beings”’ by the European Union since the enactment of the Treaty of Lisbon in 2007 [6]. However, animal sentience remains somewhat poorly defined and understood by the scientific community, let alone by lay people. A systematic review of more than 2500 peer-reviewed papers on animal sentience concluded that there was “overwhelming evidence of animal sentience” [7] (p.639). According to Broom [8], public considerations of animal sentience are influenced by factors such as how the animals are physically and cognitively similar to or different from humans. Also, attitudes to animal sentience are influenced by the role animals play in society, for example whether they are used for research, display, companionship, or food and fibre sources [8]. Acceptance of animals’ affective states and how positive emotions, such as joy and pleasure, influence the mental and physical welfare of animals fosters incremental improvements in the treatment of animals [7].

Numerous socio-demographic variables underpin attitudes to animal welfare. Previous studies of the general population indicate younger age groups show more concern for animals than older age groups [9,10,11,12]. Sex has also been found to play a strong predictive role in attitudes to animals, with many studies demonstrating that females display more concern for animals than males do [9,12,13,14,15,16,17].

With growing public awareness of and sensitivity to issues of animal welfare concern [1], veterinarians are increasingly expected to be informed about animal welfare rather than physical health alone [18,19]. However, animal welfare has not been a traditional tenet of the veterinary curriculum or profession [20]. Data on veterinarians and other animal care workers, as thought leaders, are of particular merit because they may reflect future trends among animal owners and the more general public. Many studies have looked specifically at the relationship between veterinary students’ stage-of-study and their attitudes to animal welfare [21,22]. For example, according to Paul and Podberscek [22], veterinary students in their later years of study attributed lower levels of sentience to animals than those in earlier years. Additionally, the importance students placed on the human–animal bond decreases as they progressed through their studies [23]. Some studies into veterinary professionals’ attitudes to animals have often focused on perceptions and treatment of animal pain. For example, a Canadian study from 11 years ago found that pain treatment use by veterinarians was inadequate [24]. However, a more recent study, also from Canada, found that veterinarians’ knowledge of and attitudes towards pain assessment and treatment in dogs and cats are improving [25].

People tend to hold different attitudes toward animals depending on the context in which the animals are present (for example companion versus farming) and the species involved [4,9,26]. Differences in attitudes to different species can reflect peoples’ beliefs regarding the mental abilities or the human-like qualities of the species, as well as where the species falls on the phylogenetic scale [27]. For example, people more often rate non-human primates and companion animals (e.g., cats and dogs) as having higher mental abilities than other species [28]. Coleman and colleagues [29] surveyed over 1000 Australians and found that 65% considered animal welfare important for domestic pets, 56.6% for native animals, 49.3% for farm animal welfare and 41% for animal welfare in general.

In May 2017, the University of Sydney held the 7th Robert Dixon Memorial Animal Welfare Symposium, an annual event dedicated to animal welfare. The event was titled “How does raising awareness lead to better animal welfare outcomes?” and focused on the role of awareness-raising in improving animal welfare outcomes for all animals from farmed to wild. Attendees were invited through announcements via the University’s media office and through other animal welfare groups such as the RSPCA. Attendees included people who primarily worked, studied or held a special interest or sensitivity to animal care and welfare issues. A pilot study was conducted into the attitudes to animals of this unique population of people, and the findings were compared to what is known in the literature regarding the attitudes of the general public. Moreover, it explored how the attitudes of this population clustered together.

## 2. Materials and Methods 

### 2.1. Survey Participants

The survey was voluntary and participants were registered attendees of the annual Robert Dixon Memorial Animal Welfare Symposium at the University of Sydney, which occurred in May 2017 at the University of Sydney’s Camperdown campus. A convenience sample of attendees completed the survey, with data being collected from 41 participants, the demographics of whom are outlined below in Table 1. Human ethics committee approval was provided by the University of Sydney Human Research Ethics Committee number 2015/630.

### 2.2. The Survey

A quantitative survey was designed to explore the attitudes to animals held by attendees of the two-hour animal welfare symposium. The survey was administered before the symposium began. As attendees gathered around the entrance to the conference hall where the symposium was being held, they were approached by members of the research team and asked to complete a hard-copy survey before the formal proceedings began and to hand it to a member of the research team upon entering the conference hall.

Respondents were advised that participation constituted informed consent. The survey was divided into 3 sections. Section 1 asked demographic questions, such as age, sex and occupation. Section 2 contained Paul and Podberscek’s [22] Beliefs about the Sentience of Animals Scale, which addresses participants’ beliefs in the sentience of animals, with 16 questions, assigned to four species groups (cow, pig, cat and dog). [The four specific species “cow, pig, cat and dog”, as well as the term “cow” instead of “cattle”, were the specific terms used in the original Paul and Podberscek’s [22] Beliefs about the Sentience of Animals Scale. We continued with their use for consistency]. It explored the extent to which participants believed that animals can experience sensations and emotions, e.g., “Do you think that most *Cows* can feel the sensation of *hunger*?” with participants being asked to indicate how similar they believe the four animal species are to humans in the way they think and feel. Section 3 contained Paul and Podberscek’s [22] Animal Empathy Scale which consisted of 28 statements regarding the way people feel about animals, e.g., “So long as they’re warm and well fed, I don’t think zoo animals mind being kept in cages”, with participants being asked to rate how much they agreed or disagreed with the statements. The current study differed from Paul and Podberscek’s [22] original study (that occurred almost two decades ago and surveyed veterinary students at two British universities in their first preclinical, first clinical and final years of study), in that it had been expanded to explore the attitudes of not only students, but all attendees of an animal welfare symposium.

### 2.3. Statistical Analysis

The data from the survey were collated, checked for errors and cleaned before being entered into the statistical software R statistical software which is part of the Free Software Foundation’s GNU project.

#### 2.3.1. Beliefs about the Sentience of Animals

The responses to Section 2 (beliefs about the sentience of animals) were pooled according to species (cow, pig, cat and dog) and also by emotion (hunger, pain, fear and boredom). An ordinal logistic regression was performed on the survey responses to Section 2 (beliefs about the sentience of animals) to model the effect of the demographic data (age, sex and occupation) on these variables. Type 2 ANOVA tables with likelihood ratio χ^2^ statistics for demographic values were then calculated from these models using the Anova function from R’s {car} package.

#### 2.3.2. Emotional Empathy with Animals

An ordinal logistic regression was also performed on the responses to Section 3 (emotional empathy with animals) to analyse any effect of the demographic variables (age, sex and occupation) as described for Section 2. Then, a Ward Hierarchical Clustering analysis, using Euclidian distance, was performed for the survey responses to Section 3 (emotional empathy with animals) to explore how patterns in the responses to the statements might cluster together.

## 3. Results

### 3.1. Beliefs about the Sentience of Animals

The results showed that the responses to questions from the Beliefs about the Sentience of Animals Scale were significantly related to certain demographic factors (age, sex and occupation; see Table 2 below). More specifically, for questions regarding sentience in cows, pigs and cats, age was negatively associated with questions about hunger and pain, in that older people attending an animal welfare symposium had stronger beliefs in sentience in cows, pigs and cats, regarding hunger and pain. Meanwhile, for all questions, females attending the symposium had stronger belief than males in sentience in cows, pigs and cats.

Finally, for questions regarding sentience in dogs, age was negatively associated with responses regarding pain, in that older people had stronger beliefs than younger people in pain-related sentience in dogs. Meanwhile, females displayed stronger beliefs than in sentience in dogs for all questions.

### 3.2. Emotional Empathy with Animals

The results showed that many of the participants’ responses to the 28 statements from the Animal Empathy Scale varied according to their demographic variables (age, sex, occupation; see Table 3 below).

In particular, younger and male attendees were more likely than older and female attendees to agree with the Statement 1 *(So long as they’re warm and well fed, I don’t think zoo animals mind being kept in cages)*.

Moreover, older and female attendees were more likely than younger and male attendees to agree with Statement 4 *(The thought of calves being reared in veal crates really makes me feel sad)* and Statement 5 *(Sad films about animals often leave me with a lump in my throat)*. Conversely, younger and male attendees were more likely to agree with Statement 7 *(People are too concerned about the suffering of laboratory rats and mice)*.

Female attendees were more likely than male attendees to agree with Statement 10 *(It upsets me when I see helpless old animals)*, Statement 13 *(I get very angry when I see animals being ill treated)*, Statement 15 *(Pets have a great influence on my moods)*, Statement 18 *(Seeing animals in pain upsets me)* and Statement 25 *(It makes me sad to see an animal on its own in a cage)*. Conversely, males were more likely than females to agree with Statement 16 *(Sometimes I am amazed how upset people get when an old pet dies)*.

Also, non-students, i.e., university staff and people from outside the university, were more likely than university students to agree with Statement 10 *(It upsets me when I see helpless old animals)*, Statement 13 *(I get very angry when I see animals being ill treated)*, Statement 21 *(I would always try to help if I saw a dog or puppy that seemed to be lost)* and Statement 22 *(I hate to see birds in cages where there is no room for them to fly about)*. Finally, there was an increased tendency among students towards agreement with Statement 28 *(Many people are over affectionate towards their pets)*, when compared to non-students.

Additionally, the results grouped the 28 statements in Section 3 (emotional empathy with animals) into the three clusters (outlined in Table 4 below). The three clusters suggest that participants’ responses to statements about emotionally relating to animals, the internal emotional lives of animals and the treatment of animals by humans (Cluster 1) differed from their responses to the statements about human interaction and engagement with animals (Cluster 2) and their responses to the statements about the keeping of companion animals and zoo animals (Cluster 3).

## 4. Discussion

The current pilot study sampled a rare population of individuals with clear commitment or interest to animal welfare and revealed some unexpected findings, whereby the participants’ responses to statements about emotional empathy with animals clustered together into three distinct groups based on their content. That said, consistent with previous research into socio-demographic factors, in particular sex and attitudes towards animal welfare [9,12,13,14,15,17], it also found that females hold stronger beliefs in animal sentience for all of the four focal species (cows, pigs, cats and dogs) than males do.

However, in contrast to previous research suggesting younger people show higher levels of concern for animals than older people [9,10,11,12], the current study found that older people attending an animal welfare symposium had stronger beliefs in sentience relating to hunger and pain in cows, pigs, and cats, as well as those relating to pain in dogs. Older people attending the animal welfare symposium may not be representative of older people in general and the reasons they may attend such a seminar could differ from the reasons for younger people attending. Moreover, the pilot population studied represented a unique group who mostly studied, worked, or held a special interest or sensitivity to issues of animal care and welfare and thus, the older attendees could be considered to have been within this field for longer and more strongly committed to animal welfare.

When the 28 statements regarding emotional empathy with animals were grouped, the three clusters emerged suggesting that participant responses to the statements about emotionally relating to animals, animals internal emotional lives and the treatment of animals by humans (Cluster 1) differed from their responses to the statements about human interaction and engagement with animals (Cluster 2) and their responses to the statements about the keeping of companion animals and zoo animals (Cluster 3). Interestingly, these clusters were associated with certain demographic data.

Females were more likely to agree with the statements in Cluster 1, whereas males were more likely to agree with the statements in Cluster 2. These findings could imply that females emotionally engage with animals more strongly than males do and are more likely to consider animals capable of having internal emotional lives. In contrast, males were more likely to hold pragmatic views regarding human-use and interactions with animals; for example, males were more likely to agree with Statement 1 *(So long as they’re warm and well fed, I don’t think zoo animals mind being kept in cages).*

The influence of age was also revealed in that older attendees were more likely to agree with statements in Cluster 1, specifically Statement 4 *(The thought of calves being reared in veal crates really makes me feel sad)* and Statement 5 *(Sad films about animals often leave me with a lump in my throat)*. Meanwhile, younger attendees were more likely than older attendees to agree with statements in Cluster 2, specifically Statement 1 *(So long as they’re warm and well fed, I don’t think zoo animals mind being kept in cages)* and Statement 7 *(People are too concerned about the suffering of laboratory rats and mice).* These findings suggest that older attendees of the animal welfare symposium showed more emotional engagement with animal suffering than younger attendees did. In contrast, younger attendees showed more agreement with rational, dispassionate views regarding animal use.

In a similar vein, students were more likely to agree with Statement 28 *(Many people are over affectionate towards their pets)* that was assigned to Cluster 2. In contrast, non-students i.e., university staff and external stakeholders were more likely to agree with statements in Cluster 1, in particular, Statement 21 *(I would always try to help if I saw a dog or puppy that seemed to be lost)* and Statement 22 *(I hate to see birds in cages where there is no room for them to fly about)*. These findings suggest that, compared with non-students, student attendees of the animal welfare symposium showed less empathy and assigned less importance to the role of the human-animal-bond. This is consistent with Martin, Ruby and Farnum [23] who reported that the level of importance students placed on the human-animal-bond decreased as they progressed through veterinary education. This loss of empathy as students’ progress in their course is a troubling and consistent finding which could be due to systematic practices of condemning empathy or emotional investment on the part of the practitioner as claiming it interferes with their objective assessment. As mentioned earlier, animal welfare is not a traditional tenet of the veterinary curriculum, and this remains true for the University of Sydney at which this study was undertaken. Furthermore, the heightened level of concern for animals shown by non-students could be explained by looking at the participants’ occupations, in that most participants (68%) (*n* = 28) held professional positions outside the university. For example, most worked in the animal health and welfare sector (18%), the wildlife sector or for a zoo (14%), or were a practicing veterinarian (14%).

Finally, attitudes to animals are linked to beliefs about the abilities, experiences, cognition, and sentience of animals, as well as to relationships among human and non-human animals. Attempts to raise awareness and educate the public can have a critical role in improving the treatment and welfare of animals in the human domain. Such efforts can lead to better national welfare standards, improved purchase-decisions regarding animal products, and better animal care by current and future veterinary professionals and policy makers.

## 5. Limitations

We freely acknowledge that this study was a pilot study with a small sample size of 41 respondents. This work is based on a convenience sample and therefore is not representative of the wider population. This study samples a unique population of people who attended a two-hour symposium on animal welfare held at the University of Sydney. Furthermore, the sample population shows a disproportion between males and females. However, given the veterinary profession is also heavily female biased, this is not considered surprising.

The strong associations the current results reveal are likely to be of considerable interest to those with an interest in societal shifts in concern animal welfare. They may initiate novel lines of enquiry into demographic influences on speciesism within the animal protection movement and beyond.

## 6. Conclusions

This pilot study explored the attitudes towards animal welfare of a unique population of stakeholders who worked, studied or held a strong interest in the animal care and welfare sector and therefore attended an animal welfare symposium at the University of Sydney. Its core findings are that the beliefs in animal sentience held by attendees of the animal welfare symposium varied based on the attendees demographic variables., in particular, that female attendees showed stronger belief in sentience than male attendees did. When looking at how attendees considered emotional empathy with animals, the attendees’ responses could be assigned to three clusters reflecting the internal emotional lives of animals and the treatment of animals by humans (Cluster 1), human interactions with animals (Cluster 2) and the keeping of companion and zoo animals (Cluster 3). These findings may advance the understanding of human attitudes to the welfare of non-human animals and help efforts to improve the lives of animals.

## Figures and Tables

**Table 1:** The demographics of the participants (*n* = 41).

Demographic factor		Count (Percentage)
Sex:		
Male		10 (24%)
Female		31 (76%)
Age:		
18–29 years		9 (22%)
30–39 years		9 (22%)
40–49 years		5 (12%)
50–64 years		13 (32%)
65 + years		5 (12%)
Occupation:		
University of Sydney student		11 (27%)
University of Sydney staff		2 (5%)
Other:		28 (68%)
	Working in the animal health and welfare sector	5 (18%)
	Working in the wildlife sector or for a zoo	4 (14%)
	Practicing veterinarians	4 (14%)
	Interested members of the general public	3 (11%)
	Members of industry	2 (7%)
	Working in the pet and pet food industry	1 (4%)
	Working in engineering	1 (4%)
	Did not report their profession	8 (28%)

**Table 2:** The Logistic Regression Chi-Square test χ^2^ (*p*-values) for the participants’ (*n* = 41) demographics and their response regarding beliefs about the sentience of animals by species and emotion.

		Hunger	Pain	Fear	Boredom
**Cows**	*Age*	8.263 (0.004) *	4.154 (0.042) *	3.070 (0.080)	1.721 (0.190)
	*Sex*	14.240 (< 0.001) *	12.285 (<0.001) *	8.714 (0.003) *	7.916 (0.005) *
	*Occupation*	0.061 (0.970)	0.088 (0.957)	1.644 (0.439)	2.336 (0.311)
**Pigs**	*Age*	10.922 (0.001) *	3.968 (0.046) *	2.974 (0.085)	3.476 (0.062)
	*Sex*	12.829 (< 0.001) *	10.793 (0.001) *	8.228 (0.004) *	10.549 (0.001) *
	*Occupation*	1.287 (0.525)	0.645 (0.724)	2.450 (0.294)	3.241 (0.198)
**Cats**	*Age*	10.780 (0.001) *	8.277 (0.004) *	2.054 (0.152)	0.849 (0.357)
	*Sex*	11.809 (0.001) *	13.429 (0.000) *	7.293 (0.007) *	5.307 (0.021) *
	*Occupation*	0.490 (0.783)	0.032 (0.984)	4.421 (0.110)	3.401 (0.183)
**Dogs**	*Age*	0.593 (0.441)	5.541 (0.019) *	3.331 (0.068)	0.802 (0.371)
	*Sex*	10.011 (0.002) *	13.315 (<0.001) *	4.977 (0.026) *	7.095 (0.008) *
	*Occupation*	3.339 (0.188)	0.801 (0.670)	1.264 (0.531)	2.192 (0.334)

* Denotes significant *p*-values at the 0.05 confidence level.

**Table 3:** The Logistic Regression Chi-Square test χ^2^ (*p*-values) for participants’ (*n* = 41) demographics and their responses to statements regarding emotional empathy with animals.

	Age	Sex	Occupation
1. So long as they’re warm and well fed, I don’t think zoo animals mind being kept in cages	4.752 (0.029) *	8.011 (0.005) *	7.745 (0.021) *
2. Often cats will meow and pester for food even when they are not really hungry	0.024 (0.873)	0.03 (0.864)	1.301 (0.521)
3. It upsets me to see animals being chased and killed by lions in wildlife programs on TV	2.108 (0.147)	2.672 (0.102)	0.707 (0.702)
4. The thought of calves being reared in veal crates really makes me feel sad	7.416 (0.006) *	6.246 (0.012) *	4.342 (0.114)
5. Sad films about animals often leave me with a lump in my throat	8.983 (0.003) *	4.759 (0.029) *	4.273 (0.118)
6. Animals deserve to be told off when they’re not behaving properly	1.199 (0.274)	1.474 (0.225)	2.797 (0.247)
7. People are too concerned about the suffering of laboratory rats and mice	4.823 (0.028) *	10.315 (0.001) *	1.439 (0.487)
8. People who cuddle and kiss their pets in public annoy me	1.539 (0.215)	0.045 (0.832)	5.802 (0.055)
9. A friendly purring cat almost always cheers me up	0.359 (0.549)	2.292 (0.130)	2.895 (0.235)
10. It upsets me when I see helpless old animals	0.738 (0.390)	5.151 (0.023) *	12.311 (0.002) *
11. Dogs sometimes whine and whimper for no real reason	0.062 (0.803)	0.1332 (0.716)	5.147 (0.076)
12. It makes me angry to think of things that are done to laboratory animals	0.022 (0.883)	0.734 (0.392)	2.312 (0.315)
13. I get very angry when I see animals being ill treated	0.056 (0.813)	10.545 (0.001) *	8.038 (0.018) *
14. It is silly to become too attached to one’s pets	0.441 (0.507)	1.296 (0.255)	4.237 (0.120)
15. Pets have a great influence on my moods	3.540 (0.060)	5.472 (0.019) *	5.928 (0.052)
16. Sometimes I am amazed how upset people get when an old pet dies	0.013 (0.910)	4.362 (0.037) *	4.118 (0.128)
17. It’s silly to worry about how farm animals feel	1.632 (0.201)	3.790 (0.052)	5.218 (0.074)
18. Seeing animals in pain upsets me	0.177 (0.674)	7.607 (0.006) *	4.338 (0.114)
19. People often make too much of the feelings and sensitivities of animals	0.546 (0.460)	2.340 (0.126)	2.398 (0.302)
20. I find it irritating when dogs try to greet me by jumping up and licking me	0.925 (0.336)	0.005 (0.942)	0.355 (0.837)
21. I would always try to help if I saw a dog or puppy that seemed to be lost	0.941 (0.332)	0.545 (0.461)	11.835 (0.003) *
22. I hate to see birds in cages where there is no room for them to fly about	0.373 (0.542)	1.766 (0.184)	8.661 (0.013) *
23. It upsets me to see farm animals in lorries going to slaughter	0.484 (0.487)	2.972 (0.085)	4.453 (0.108)
24. I enjoy feeding scraps of food to the birds	0.553 (0.457)	0.007 (0.935)	2.820 (0.244)
25. It makes me sad to see an animal on its own in a cage	3.354 (0.067)	5.948 (0.015) *	4.952 (0.084)
26. I get annoyed by dogs that howl and bark when they are left alone	0.240 (0.624)	3.812 (0.051)	3.072 (0.215)
27. I hate seeing pictures of animals used in scientific experiments	0.320 (0.572)	0.506 (0.477)	5.368 (0.068)
28. Many people are over affectionate towards their pets	1.078 (0.299)	0.381 (0.537)	7.985 (0.018) *

* Denotes significant *p*-values at the 0.05 confidence level.

**Table 4:** The Ward Hierarchical Clustering analysis for participants’ responses (*n* = 41) regarding emotional empathy with animals.

**Cluster 1**
4. The thought of calves being reared in veal crates really makes me feel sad
5. Sad films about animals often leave me with a lump in my throat
9. A friendly purring cat almost always cheers me up
10. It upsets me when I see helpless old animals
12. It makes me angry to think of things that are done to laboratory animals
13. I get very angry when I see animals being ill treated
15. Pets have a great influence on my moods
18. Seeing animals in pain upsets me
21. I would always try to help if I saw a dog or puppy that seemed to be lost
22. I hate to see birds in cages where there is no room for them to fly about
23. It upsets me to see farm animals in lorries going to slaughter
25. It makes me sad to see an animal on its own in a cage
27. I hate seeing pictures of animals used in scientific experiments
**Cluster 2**
1. So long as they’re warm and well fed, I don’t think zoo animals mind being kept in cages
7. People are too concerned about the suffering of laboratory rats and mice
11. Dogs sometimes whine and whimper for no real reason
14. It is silly to become too attached to one’s pets
16. Sometimes I am amazed how upset people get when an old pet dies
17. It’s silly to worry about how farm animals feel
19. People often make too much of the feelings and sensitivities of animals
28. Many people are over affectionate towards their pets
**Cluster 3**
2. Often cats will meow and pester for food even when they are not really hungry
3. It upsets me to see animals being chased and killed by lions in wildlife programs on TV
6. Animals deserve to be told off when they’re not behaving properly
8. People who cuddle and kiss their pets in public annoy me
20. I find it irritating when dogs try to greet me by jumping up and licking me
24. I enjoy feeding scraps of food to the birds
26. I get annoyed by dogs that howl and bark when they are left alone
